# Orienting the causal relationship between imprecisely measured traits using GWAS summary data

**DOI:** 10.1371/journal.pgen.1007081

**Published:** 2017-11-17

**Authors:** Gibran Hemani, Kate Tilling, George Davey Smith

**Affiliations:** MRC Integrative Epidemiology Unit (IEU) at the University of Bristol, School of Social and Community Medicine, Bristol, United Kingdom; Harvard University, UNITED STATES

## Abstract

Inference about the causal structure that induces correlations between two traits can be achieved by combining genetic associations with a mediation-based approach, as is done in the causal inference test (CIT). However, we show that measurement error in the phenotypes can lead to the CIT inferring the wrong causal direction, and that increasing sample sizes has the adverse effect of increasing confidence in the wrong answer. This problem is likely to be general to other mediation-based approaches. Here we introduce an extension to Mendelian randomisation, a method that uses genetic associations in an instrumentation framework, that enables inference of the causal direction between traits, with some advantages. First, it can be performed using only summary level data from genome-wide association studies; second, it is less susceptible to bias in the presence of measurement error or unmeasured confounding. We apply the method to infer the causal direction between DNA methylation and gene expression levels. Our results demonstrate that, in general, DNA methylation is more likely to be the causal factor, but this result is highly susceptible to bias induced by systematic differences in measurement error between the platforms, and by horizontal pleiotropy. We emphasise that, where possible, implementing MR and appropriate sensitivity analyses alongside other approaches such as CIT is important to triangulate reliable conclusions about causality.

## Author summary

Understanding the causal relationships between pairs of traits is crucial for unravelling the causes of disease. To this end, results from genome-wide association studies are valuable because if a trait is known to be influenced by a genetic variant then this knowledge can be used to test the trait’s causal influences on other traits and diseases. Here we discuss scenarios where the nature of the genetic association with the causal trait can lead existing causal inference methods to give the wrong direction of causality. We introduce a new method that can be applied to summary level data and is potentially less susceptible to problems such as measurement error, and apply it to evaluate the causal relationships between DNA methylation levels and gene expression. While our results show that DNA methylation is more likely to be the causal factor, we point out that is it crucial to acknowledge that systematic differences in measurement error between the platforms could influence such conclusions.

## Introduction

Observational measures of the human phenome are growing ever more abundant, but using these data to make causal inference is notoriously susceptible to many pitfalls, with basic regression-based techniques unable to distinguish a true causal association from reverse causation or confounding [1–3]. In response to this, the use of genetic associations to instrument traits has emerged as a technique for improving the reliability of causal inference in observational data, and with the coincident rise in genome-wide association studies it is now a prominent tool that is applied in several different guises [3–6]. However, shifting from observational associations to instrumentation does require more (often untestable) assumptions, and potential pitfalls remain. One that is often neglected is the influence of non-differential measurement error on the reliability of causal inference.

Measurement error is the difference between the measured value of a quantity and its true value. This study focuses specifically on non-differential measurement error where all strata of a measured variable have the same error rate, which can manifest as changes in scale or measurement imprecision (noise). Such variability can arise through a whole plethora of mechanisms, which are often specific to the study design and difficult to avoid [7, 8]. Array technology is now commonly used to obtain high throughput phenotyping at low cost, but comes with the problem of having imperfect resolution, for instance methylation levels as measured by the Illumina450k chip are prone to have some amount of noise around the true value due to imperfect sensitivity [9, 10]. Relatedly, if the measurement of biological interest is the methylation level in a T cell, then measurement error of this value can be introduced by using methylation levels from whole blood samples because the measured value will be an assay of many cell types [11].

Measurement error will of course arise in other types of data too. For example when measuring BMI one is typically interested in using this as a proxy for adiposity, but it is clear that the correlation between BMI and underlying adiposity is not perfect [12], leading to the problem that phenotypes may be imprecisely defined. A similar problem of biological misspecification is unavoidable in disease diagnosis, and measuring behaviour such as smoking or diet is notoriously difficult to do accurately. Measurement error can also be introduced after the data have been collected, for example the transformation of non-normal data for the purpose of statistical analysis will lead to a new variable that will typically incur both changes in scale and imprecision (noise) compared to the original variable. The sources of measurement error are not limited to this list [8], and its impact has been explored in the epidemiological literature extensively [13, 14]. Given the near-ubiquitous presence of measurement error in phenomic data it is vital to understand its impact on the tools we use for causal inference.

An established study design that can provide information about causality is randomisation. Given the hypothesis that trait A (henceforth referred to as the exposure) is causally related to trait B (henceforth referred to as the outcome), randomisation can be employed to assess the causal nature of the association by randomly splitting the sample into two groups, subjecting one group to the exposure and treating the other as a control. The association between the exposure and the outcome in this setting provides a robust estimate of the causal relationship. This provides the theoretical basis behind randomised control trials, but in practice randomisation is often difficult or impossible to implement in an experimental context due to cost, scale or inability to manipulate the exposure. The principle, however, can be employed in extant observational data through the use of genetic variants associated with the exposure (instruments), where the inheritance of an allele serves as a random lifetime allocation of differential exposure levels [15, 16]. Two statistical approaches to exploiting the properties of genetic instruments are widely used: mediation-based approaches and Mendelian randomisation (MR).

Mediation-based approaches employ genetic instruments (typically single nucleotide polymorphisms, SNPs) to orient the causal direction between the exposure and the outcome. If a SNP is associated with an exposure, and the exposure is associated with some outcome, then it logically follows that in this simple three-variable scenario the estimated direct influence of the SNP on the outcome will be zero when conditioning on the exposure. Here, the exposure completely mediates the association between the SNP and the outcome, providing information about the causal influence of the exposure on the outcome. This forms the basis of a number of methods such as genetical genomics [17], the regression-based causal inference test (CIT) [4, 18], a structural equation modelling (SEM) implementation in the NEO software [5], and various other methods including Bayesian approaches [6]. They have been employed by a number of recent publications that make causal inferences in large scale ‘omics datasets [6, 19–23].

MR can be applied to the same data—phenotypic measures of the exposure and the outcome variables and a genetic instrument for the exposure—but the genetic instrument is employed in a subtly different manner. Here the SNP is used as a surrogate for the exposure. Assuming the SNP associates with the outcome only through the exposure, the causal effect of the exposure on the outcome can be estimated by scaling the association between the SNP and the outcome by the association between the SNP and the exposure. Though difficult to test empirically, this assumption can be relaxed in various ways when multiple instruments are available for a putative exposure [24, 25] and a number of sensitivity tests are now available to improve reliability [26]. Additionally, if valid genetic instruments are known for both traits of interest then MR can be performed in both directions (bi-directional MR), testing the influence of one trait on the other and vice versa, to infer the causal direction between the two phenotypes [27, 28].

**Fig 1:**
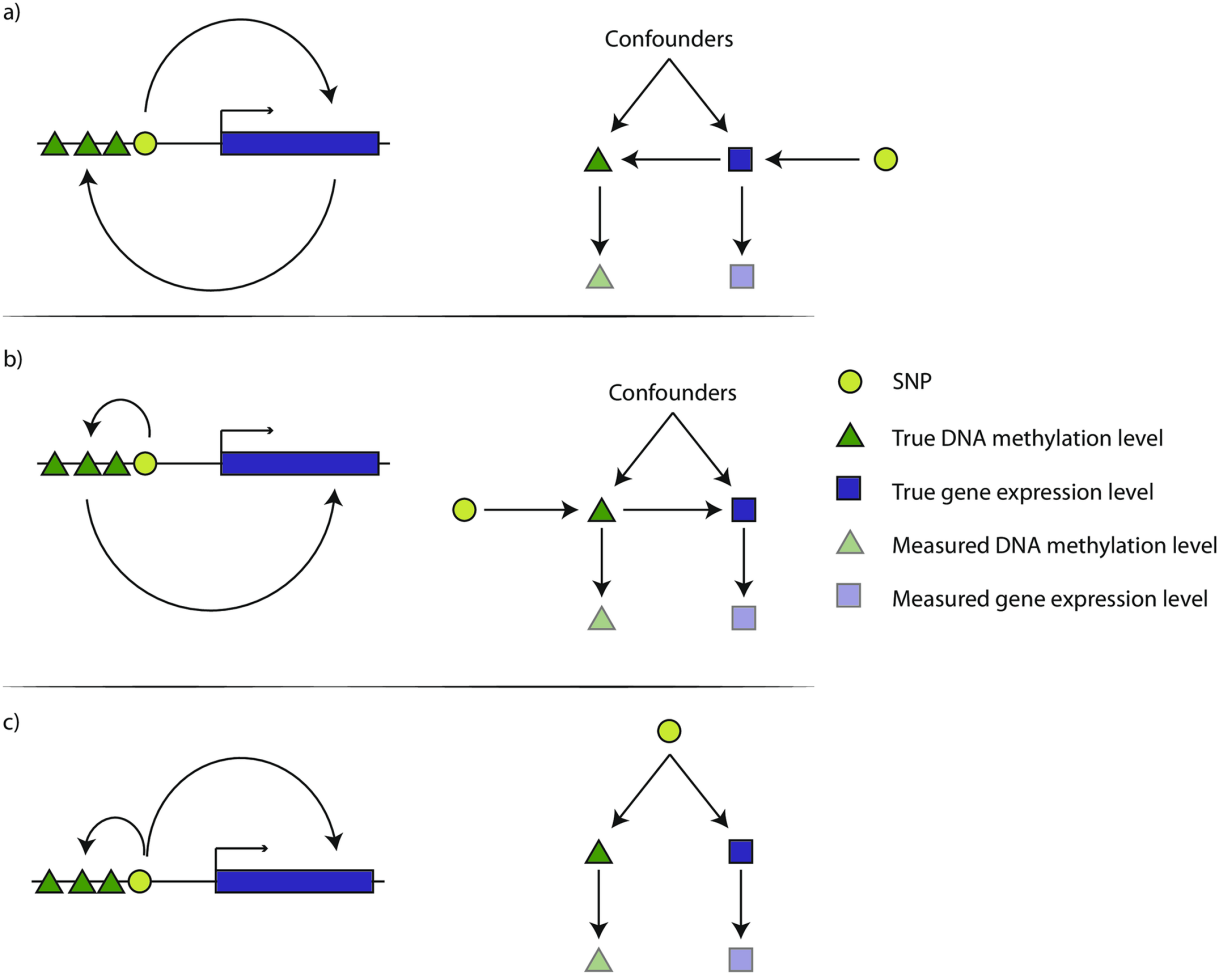
Gene expression levels (blue blocks) and DNA methylation levels (green triangles) may be correlated but the causal structure is unknown. If a SNP (yellow circle) is associated with both DNA methylation and gene expression levels then it can be used as an instrument, but there are three basic competing models for these variables. The causal inference test (CIT) attempts to distinguish between them. a) Gene expression causes methylation. The left figure shows that the SNP influences gene expression levels that in turn influence methylation levels. The right figure shows the directed acyclic graph that represents this model. Faded symbols represent the measured values whereas solid symbols represent the true values. b) The same as in A, except the causal direction is from DNA methylation to Gene expression. c) A model of confounding, where gene expression and DNA methylation are not causally related, but the SNP influences them each through separate pathways or a confounder.

By utilising genetic instruments in different ways, mediation-based analysis and MR models have properties that confer some advantages and some disadvantages for reliable causal inference. In the CIT framework (described fully in the Methods) for example, the test statistic is different if you test for the exposure causing the outcome or the outcome causing the exposure, allowing the researcher to infer the direction of causality between two variables by performing the test in both directions and choosing the model with the strongest evidence. The CIT also has the valuable property of being able to distinguish between several putative causal graphs that link the traits with the SNP (Fig 1). Such is not the case for MR, where in order to infer the direction of causality between two traits the instrument must have its most proximal link with the exposure, associating with the outcome only through the exposure.

Assuming biological knowledge of genetic associations can be problematic because if there exists a putative association between two variables, with the SNP being robustly associated with each, it can be difficult to determine which of the two variables is subject to the primary effect of the SNP (i.e. for which of the two variables is the SNP a valid instrument? Fig 1). By definition, we expect that if the association is causal then a SNP for the exposure will be associated with the outcome, such that if the researcher erroneously uses the SNP as an instrument for the outcome then they are likely to see an apparently robust causal association of outcome on exposure. Genome-wide association studies (GWASs) that identify genetic associations for complex traits are, by design, hypothesis free and agnostic of genomic function, and it often takes years of follow up studies to understand the biological nature of a putative GWAS hit [29]. If multiple instruments are available for an hypothesised exposure, which is increasingly typical for complex traits that are analysed in large GWAS consortia, then techniques can be applied to mitigate these issues [16]. But these techniques cannot always be applied in the case of determining causal directions between ’omic measures where typically only one cis-acting SNP is known. For example if a DNA methylation probe is associated with expression of an adjacent gene, then is a cis-acting SNP an instrument for the DNA methylation level, or the gene expression level (Fig 1)?

MR has some important advantages over the mediation-based approaches. First, the mediation-based approaches require that the exposure, outcome and instrumental variables are all measured in the same data, whereas recent extensions to MR circumvent this requirement, allowing causal inference to be drawn when exposure variables and outcome variables are measured in different samples [30]. This has the crucial advantage of improving statistical power by allowing analysis in much larger sample sizes, and dramatically expands the breadth of possible phenotypic relationships that can be evaluated [26]. Second, the mediation-based approach of adjusting the outcome for the exposure to nullify the association between the SNP and the outcome is affected by unmeasured confounding of the exposure and outcome. This is because adjusting the outcome by the exposure induces a collider effect between the SNP and outcome [31], and in order to fully abrogate this association one must also adjust for all (hidden or otherwise) confounders. MR does not suffer from this problem because it does not test for association through adjustment. Third, when MR assumptions are satisfied the method is robust to there being measurement error in the exposure variable [32]. Indeed instrumental variable (IV) analysis was in part initially introduced as a correction for measurement error in the exposure [33], whereas it has been noted that both classic mediation-based analyses [13, 14, 34, 35] and mediation-based methods that use instrumental variables [36, 37] are prone to be unreliable in its presence.

Using theory and simulations we show how non-differential measurement error in phenotypes can lead to unreliable causal inference in the mediation-based CIT method. Though we only examine the CIT method in detail, we believe that attempting to adjust for mediating variables to make causal inference is susceptible to problems, which can be generalised to other mediation-based methods. We then present an extension to MR that allows researchers to ascertain the causal direction of an association even when the biology of the instruments are not fully understood, and also a metric to evaluate the sensitivity of the result of this extension to measurement error. Finally, to demonstrate the potential impact of measurement error we apply this method to infer the direction of causation between DNA methylation levels and gene expression levels. Our analyses highlight that because these different causal inference techniques have varying strengths and weaknesses, triangulation of evidence from as many sources as possible should be practiced in causal inference [38].

### Model

We model a system whereby some exposure *x* has a causal influence *β*_*x*_ on an outcome *y* such that

$$y=\alpha_{x}+\beta_{x}x+\epsilon_{x}$$

In addition, the exposure is influenced by a SNP *g* with an effect of *β*_*g*_ such that

$$x=\alpha_{g}+\beta_{g}g+\epsilon_{g}$$

The α_*_ terms represent intercepts, and henceforth can be ignored. The ϵ_*_ terms denote random error, assumed independently and normally distributed with mean zero. Mediation-based analyses that test whether *x* causally relates to *y* rely on evaluating whether the influence of *g* on *y* can be accounted for by conditioning on *x*, such that

$$cov(g,y-\hat{y})=0$$

where $\hat{y}={\hat{\beta }}_{x}x$ and assuming no intercept $y-\hat{y}=\epsilon_{x}$. MR analysis estimates the causal influence of *x* on *y* by using the instrument as a proxy for *x*, such that

$$\begin{array}{ccc} \hat{x} & = & {\hat{\beta }}_{g}g\\ y & = & \beta_{MR}\hat{x}+\epsilon_{MR} \end{array}$$

where *β*_*MR*_ ≠ 0 denotes the existence of causality, and *β*_*MR*_ is an estimate of the causal effect.

Measurement error of an exposure can be modeled as a transformation of the true value (*x*) that leads to the observed value, *x*_*o*_ = *f*(*x*). For example, following Pierce and VanderWeele [32] we can define

$$f\left(x\right)=\alpha_{mx}+\beta_{mx}x+\epsilon_{mx}$$

where *α*_*mx*_ and *β*_*mx*_ influence the error in the measurement of *x* by altering its scale, and *ϵ*_*mx*_ represents the imprecision (or noise) in the measurement of *x*. Measurement imprecision can represent imprecise measurement due to limits on sensitivity of measuring equipment, or arise because of phenotypes being imprecisely defined. The same model of measurement error can be applied to the outcome variable *y*.

In this study we assume there is no measurement error in the SNP. Common genetic variants are typically less susceptible to measurement error due to strict quality control procedures prior to genome wide association studies. Any non-differential measurement error that might be present (either because the SNP is poorly typed or because the SNP is not in complete linkage disequilibrium with the causal variant) will reduce power in MR but will not incur bias [3, 13, 32]. We also assume that measurement error in the exposure and the outcome are uncorrelated.

## Results

### Mediation-based causal inference under measurement error

In the causal inference test (CIT), the 4th condition (see Methods) employs mediation for causal inference, and can be expressed as $cov(g,y-\hat{y})=0$, where $\hat{y}={\hat{\alpha }}_{x}+{\hat{\beta }}_{x}x_{o}$. When measurement error in scale and imprecision is introduced, such that *y*_*o*_ is the measured value of *y*, it can be shown using basic covariance properties (S1 Text) that

$$\begin{array}{ccc} cov(g,y-\hat{y}) & = & cov\left(g,y_{o}\right)-cov\left(g,{\hat{y}}_{o}\right)\\ & = & \beta_{my}\beta_{g}\beta_{x}var\left(g\right)-D\beta_{my}\beta_{g}\beta_{x}var\left(g\right) \end{array}$$

where

$$D=\frac{\beta_{mx}^{2}var\left(x\right)}{\beta_{mx}^{2}var\left(x\right)+var\left(\epsilon_{mx}\right)}$$

Thus an observational study will find $cov(g,y_{o}-\hat{y_{o}})=0$ when the true model is causal only when *D* = 1. Therefore, if there is any measurement error that incurs imprecision in *x* (i.e. *var*(*ϵ*_*mx*_) ≠ 0) then there will remain an association between *g* and *y*_*o*_|*x*_*o*_, which is in violation of the the 4th condition of the CIT. Note that scale transformation of *x* or *y* without any incurred imprecision is insufficient to lead to a violation of the test statistic assumptions, and henceforth mention of measurement error will relate to imprecision unless otherwise stated.

**Fig 2:**
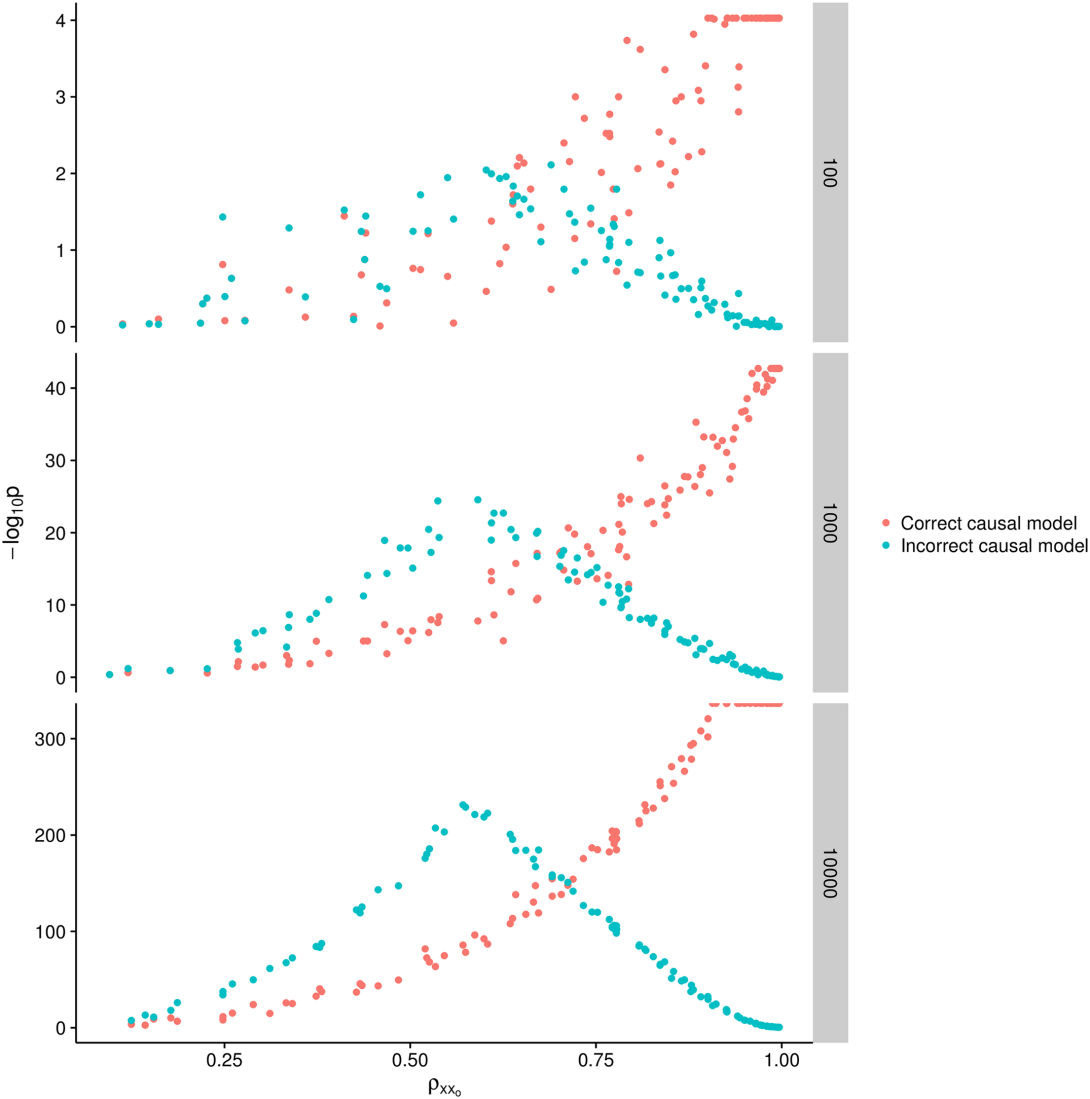
The CIT was performed on simulated variables where the exposure influenced the outcome and the exposure was instrumented by a SNP. The test statistic from CIT when testing if the exposure caused the outcome (the true model) is in red, and the test for the outcome causing the exposure (false model) is in green. Rows of plots represent the sample sizes used for the simulations. As measurement imprecision increases (decreasing values on x-axis) the test statistic for the incorrect model gets stronger and the test statistic for the correct model gets weaker.

We performed simulations to verify that this problem does arise using the CIT method. Fig 2 shows that when there is no measurement error in the exposure or outcome variables (*ρ*_*x*,*x*_*o*__ = *ρ*_*y*,*y*_*o*__ = 1) the CIT is reliable in identifying the correct causal direction. However, as measurement error increases in the exposure variable, eventually the CIT is more likely to infer a robust causal association in the wrong direction. Also of concern here is that increasing sample size does not solve the issue, indeed it only strengthens the apparent evidence for the incorrect inference.

### Using MR Steiger to infer the direction of causality

If we do not know whether the SNP *g* has a primary influence on *x* or *y* then CIT can attempt to infer the causal direction. Though bi-directional MR can be used to orient causal directions [27], this requires knowledge of a valid instrument for each trait, and we were motivated to develop the MR Steiger method that could operate on summary data to orient the direction of causality using the same conditions as the CIT, where the underlying biology of a single SNP is not fully understood. We go on to explore the scenarios in which the method is likely to return the correct or incorrect causal directions.

**Fig 3:**
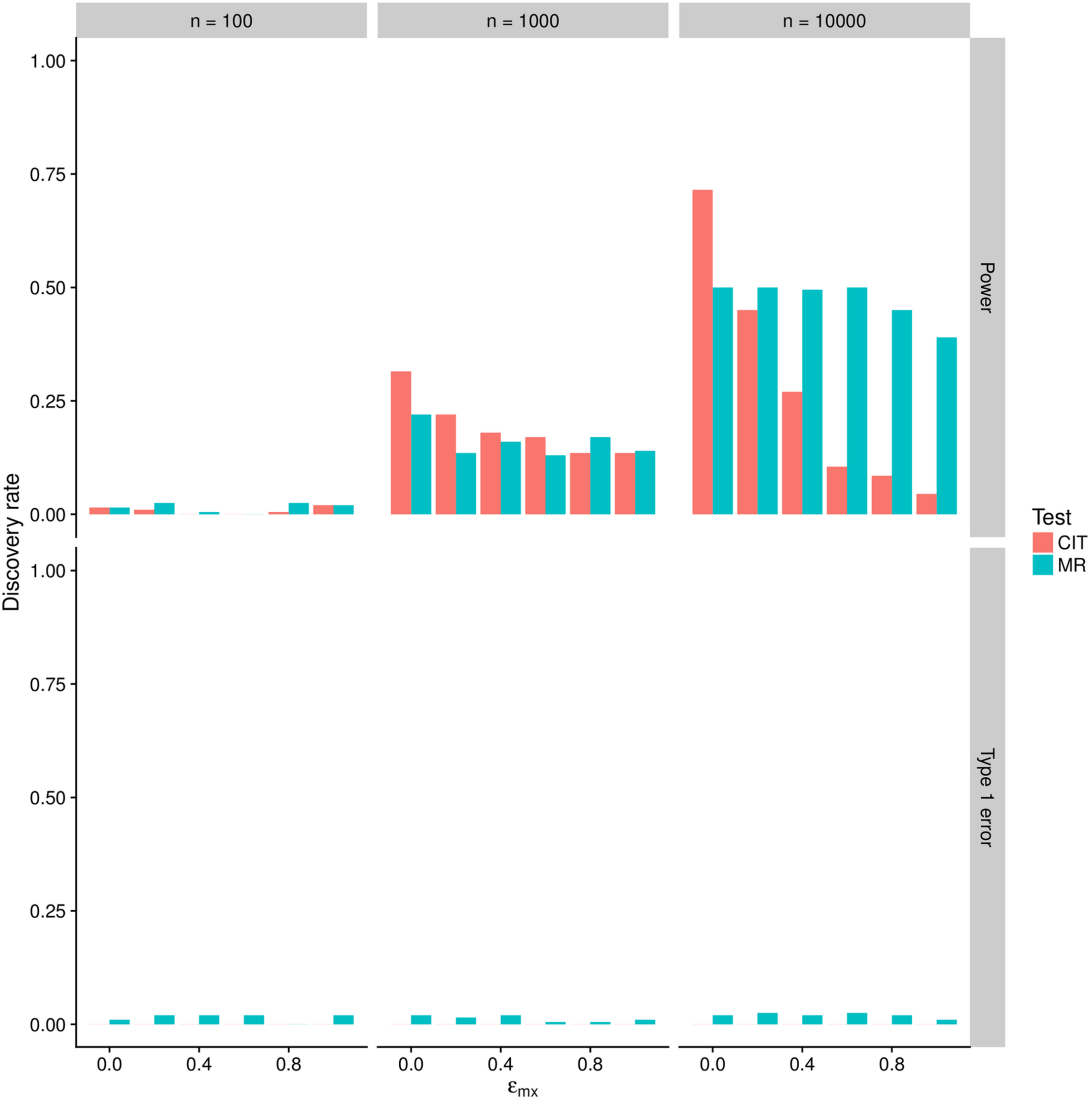
Outcomes were simulated to be unrelated to the exposure (bottom plot, showing false positive rates on the y-axis) or causally influenced by the exposure (top plot, showing true positive rates on the y-axis) with varying degrees of measurement imprecision applied to the exposure variable (x axis). Results for MR and CIT were compared for varying sample sizes (columns of boxes).

We performed simulations to compare the power and type 1 error rates of MR and CIT in detecting a causal association between simulated variables under different levels of imprecision simulated in the exposure. Comparing the performance of methods with different sets of assumptions can be difficult, but a basic comparison is shown in Fig 3. We observe that the CIT is more conservative under the null model of no association owing to the omnibus test statistic comprising several statistical tests. The FDR using a p-value threshold of 0.05 appears to be close to zero, whereas for the MR Steiger method the FDR is around 0.05. Using the same p-value thresholds to declare significance in the non-null simulations, the general trend appears to be that the CIT power reduces as measurement error in the exposure increases more steeply than that of the MR Steiger method.

**Fig 4:**
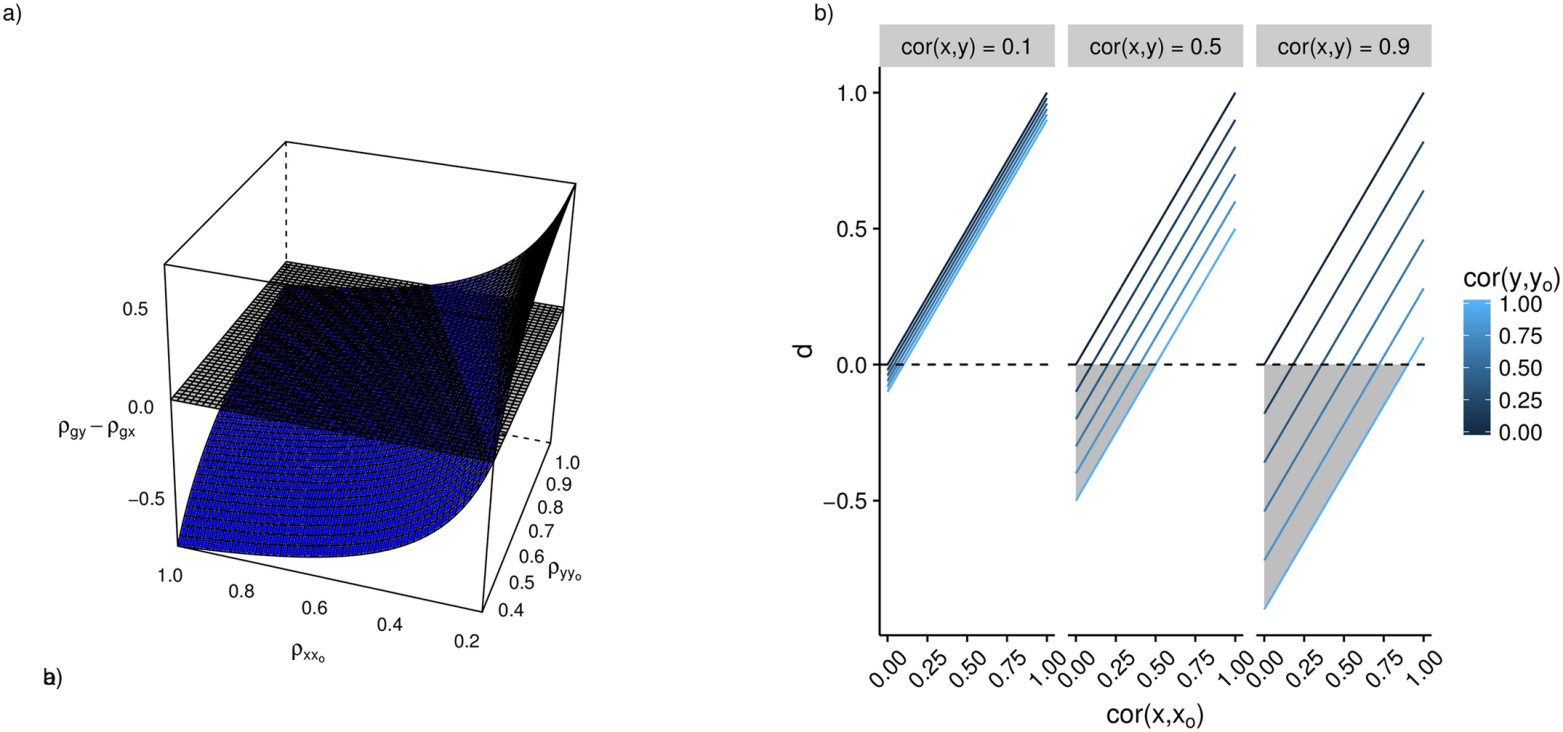
a) We can predict the values the MR Steiger test would take (z-axis) for different potential values of measurement error (x and y axes), drawn here as the blue surface. When *ρ*_*g*,*y*_ > *ρ*_*g*,*x*_, as denoted by the range of values where the blue surface is above the black plane, those values of measurement error lead to our observed MR Steiger test inferring the wrong causal direction. Where the blue surface lies below the black plane, these measurement error values support the inferred causal direction of X to Y. A measure of reliability, therefore, is the ratio of the negative and positive volumes of the total space bound by the blue and black surfaces, $R=\frac{V_{z\geq 0}}{-V_{z<0}}$. In this case, where $\rho_{g,x}^{2}=0.01$ and $\rho_{x,y}^{2}=0.1$, the *R* = 4.40, which means that 4.40 times as much of the possible measurement error values are in support of the *x* → *y* direction of causality than *y* → *x*. b) Plots depicting the parameter space in which the function *d* = *cor*(*x*, *x*_*O*_) − *cor*(*x*, *y*)*cor*(*y*, *y*_*O*_) is negative. When *d* is negative the MR Steiger test is liable to infer the wrong direction of causality. Shaded regions show the parameter space where *d* is negative. The graph shows that for the majority of the parameter space of the function, *d* is positive, especially where causal relationships are relatively weak.

For a particular association, it is of interest to identify the range of possible measurement error values for which the method will give results that agree or disagree with the empirically inferred causal direction (Fig 4a, S2 Text). This metric can be used to evaluate the reliability of MR Steiger test.

We show that in the presence of measurement imprecision, *d* = *ρ*_*x*,*x*_*o*__ − *ρ*_*x*,*y*_*ρ*_*y*,*y*_*o*__ (S2 Text) determines the range of parameters around which the MR Steiger test is liable to provide the wrong direction of causality (*i.e*. if *d* > 0 then the MR Steiger test is likely to be correct about the causal direction). Fig 4b shows that when there is no measurement error in *x*, the MR Steiger test is unlikely to infer the wrong direction of causality even if there is measurement error in *y*. It also shows that in most cases where *x* is measured with error, especially when the causal effect between *x* and *y* is not very large, the sensitivity of the MR Steiger test to measurement error is relatively low.

Unmeasured confounding between the exposure and outcome can also give rise to problems with the MR Steiger approach (S3 Text). The relationship between unmeasured confounding and causal orientation is complex across the parameter space of possible confounding values (S2 Fig). Based on the range of parameter values that we explored, when the magnitude of the observational variance explained between the exposure and the outcome is below 0.2 the MR Steiger method is unlikely to return the incorrect causal direction due to unmeasured confounding.

### Comparison of CIT and MR Steiger for obtaining the correct direction of causality

We used simulations to explore the performance of the MR Steiger approach in comparison to CIT for different levels of measurement error. The performance was compared in terms of the rate at which evidence of a causal relationship is obtained for the correct direction of causality, and the rate at which evidence of a causal relationship is obtained where the reported direction of causality is incorrect. Simulations were performed for two models, one for a “causal model” where there was a causal relationship between *x* and *y*; and one for a “non-causal model” where *x* and *y* were not causally related, but had a confounded association induced by the SNP *g* influencing *x* and *y* independently.

**Fig 5:**
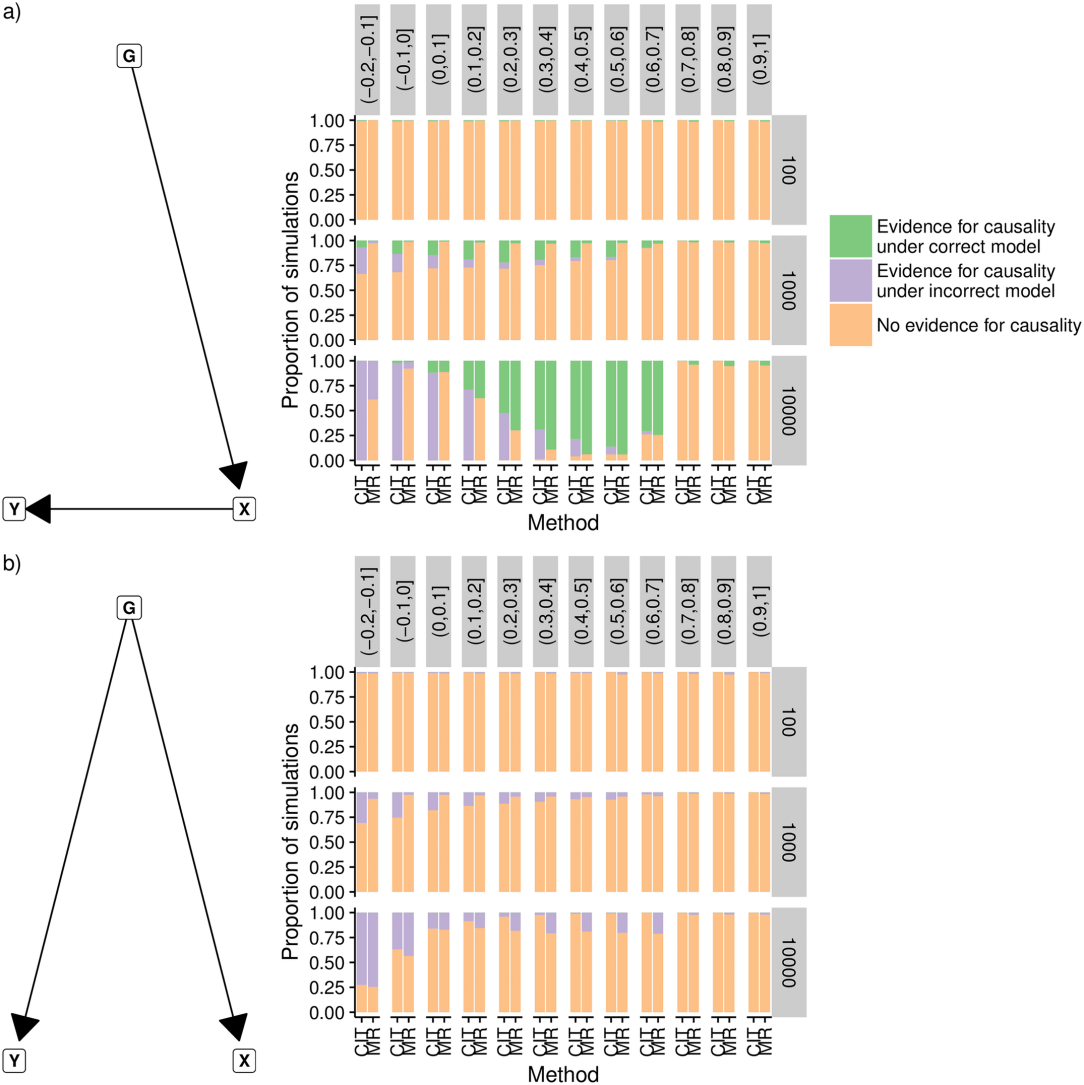
a) Outcome *y* was simulated to be caused by exposure *x* as shown in the graph, with varying degrees of measurement error applied to both. CIT and MR were used to infer evidence for causality between the exposure and outcome, and to infer the direction of causality. The columns of graphs denote intervals for he value of *d* = *ρ*_*x*,*x*_*o*__ − *ρ*_*x*,*y*_*ρ*_*y*,*y*_*o*__, such that when *d* is negative we expect the MR Steiger test to be more likely to be wrong about the direction of causality. Rows of graphs represent the sample size used in the simulations. For the CIT method, outcome 1 denoted evidence for causality with correct model, outcomes 2 or 3 denoted evidence for causality with incorrect model, and outcome 4 denoted no evidence for causality. b) As in (a) except the simulated model was non-causal, and a genetic confounder induces an association between *x* and *y*. Neither CIT nor MR are able to identify this model, so any significant associations in MR are deemed to be incorrect, while outcomes 1 or 2 for the CIT are deemed to be incorrect.

Fig 5a shows that, for the “causal model”, the MR analysis is indeed liable to infer the wrong direction of causality when *d* < 0, and that this erroneous result is more likely to occur with increasing sample size. However, the CIT is in general more fallible to reporting a robust causal association for the wrong direction of causality. When *d* > 0 we find that in most cases the MR Steiger method has greater power to obtain evidence for causality than CIT, and always obtains the correct direction of causality. The CIT, unlike the MR Steiger test, is able to distinguish the “non-causal model” from the “causal model” (Methods, Fig 5b), but it is evident that measurement error will often lead the CIT to identify the causal model as true, when in fact the underlying model is this non-causal model.

### The causal relationship between gene expression and DNA methylation levels

**Fig 6:**
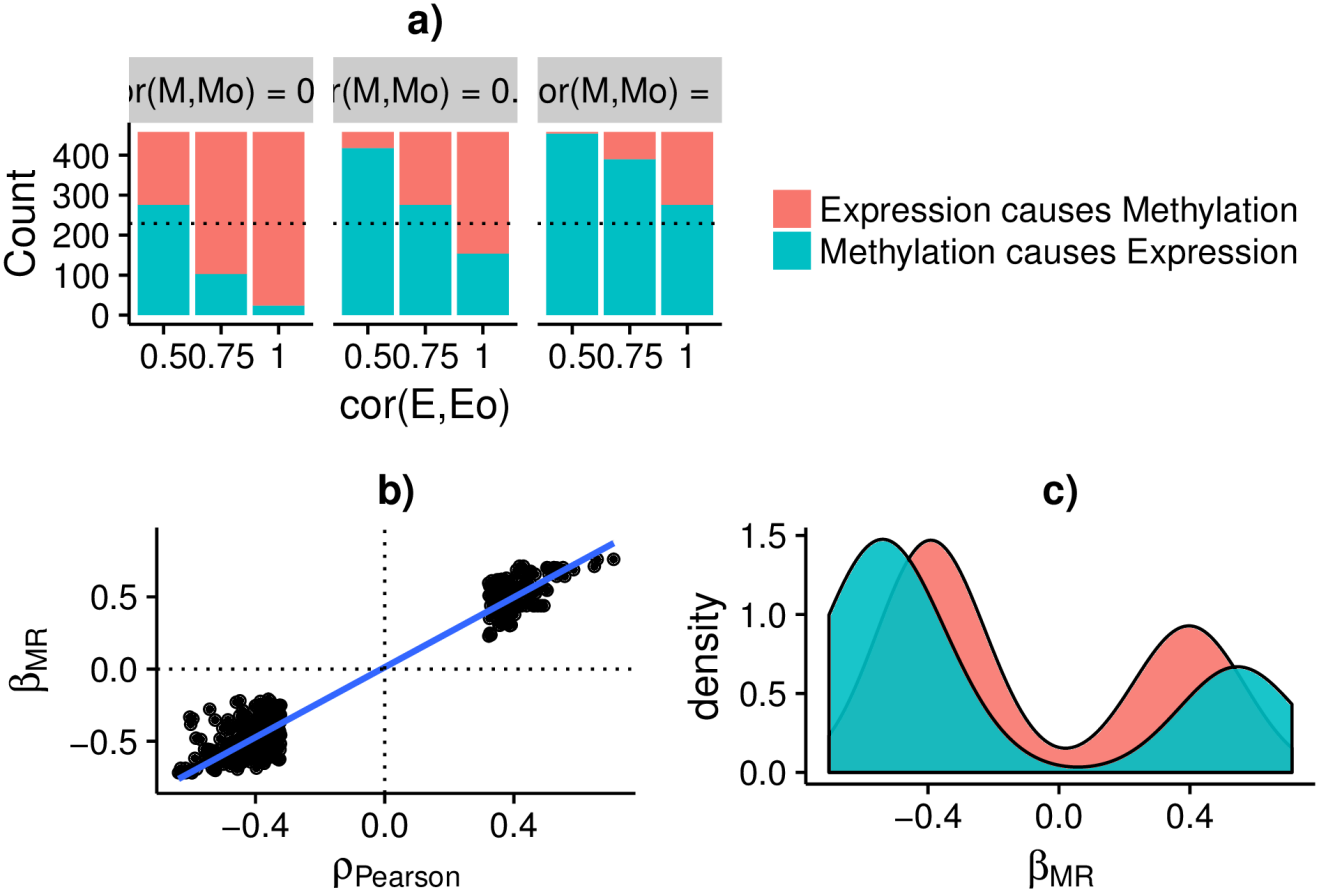
Using 458 putative associations between DNA methylation and gene expression we used the MR Steiger test to infer the direction of causality between them. a) The rightmost bar shows the proportion of associations for each of the two possible causal directions (colour key) assuming no measurement error in either gene expression or DNA methylation levels. The proportions change when we assume different levels of measurement error in gene expression levels (x-axis) or DNA methylation levels (columns of boxes). If there is systematically higher measurement error in one platform than the other it will appear to be less likely to be the causal factor. b) The relationship between the Pearson correlation between DNA methylation and gene expression levels (x-axis) and the causal estimate (scaled to be in standard deviation units, y-axis). c) Distribution of estimated causal effect sizes, stratified into associations inferred to be due to DNA methylation causing expression (blue) and expression causing DNA methylation (red).

We used the MR Steiger test to infer the direction of causality between DNA methylation and gene expression levels between 458 putative associations. We found that the causal direction commonly goes in both directions (Fig 6a), but assuming no or equal measurement error, DNA methylation levels were the predominant causal factor (*p* = 1.3 × 10^−5^). The median reliability (R) of the 458 tests was 3.92 (5%-95% quantiles 1.08–37.11). We then went on to predict the causal directions of the associations for varying levels of systematic measurement error for the different platforms. Fig 6a shows that the conclusions about the direction of causality between DNA methylation and gene expression are very sensitive to measurement error. We made a strong assumption that either methylation influenced gene expression or vice versa, but it is certainly possible that the SNP is solely or additionally influencing some other trait that confounds the association between gene expression and DNA methylation.

We performed two sample MR [30] for each association in the direction of causality inferred by the Stieger test. We observed that the sign of the MR estimate was generally in the same direction as the Pearson correlation coefficient reported by Shakhbazov et al [39] (Fig 6b). There was a moderate correlation between the absolute magnitudes of the causal correlation and the observational Pearson correlation (r = 0.45). Together these inferences suggest that even in estimating associations between ‘omic’ variables, which are considered to be low level phenotypes, it is important to use causal inference methods over observational associations to infer causal effect sizes.

We also observed that for associations where methylation caused gene expression the causal effect was more likely to be negative than for the associations where gene expression caused methylation (OR = 0.61 (95% CI 0.36–1.03), Fig 6c), suggesting that reducing methylation levels at a controlling CpG typically leads to increased gene expression levels, consistent with expectation [40].

## Discussion

Researchers are often confronted with the problem of making causal inferences using a statistical framework on observational data. In the epidemiological literature issues of measurement error in mediation analysis are relatively well explored [41]. Our analysis extends this to related methods such as CIT that are used in predominantly ’omic data. These methods are indeed susceptible to the same problem as standard mediation based analysis, and specifically we show that as measurement error in the (true) exposure variable increases, CIT is likely to have reduced statistical power, and liable to infer the wrong direction of causality. We also demonstrate that, though unintuitive, increasing sample size does not resolve the issue, rather it leads to more extreme p-values for the model that predicts the wrong direction of causality.

Under many circumstances a practical solution to this problem is to use Mendelian randomisation instead of methods such as the CIT or similar that are based on mediation. Inferring the existence of causality using Mendelian randomisation is robust in the face of measurement error and, if the researcher has knowledge about the biology of the instrument being used in the analysis, can offer a direct solution to the issues that the CIT faces. This assumption is often reasonable, for example SNPs are commonly used as instruments when they are found in genes with known biological relevance for the trait of interest. But on many occasions, especially in the realm of ’omic data, this is not the case, and methods based on mediation have been valuable in order to be able to both ascertain if there is a causal association and to infer the direction of causality. Here we have described a simple extension to MR which can be used as an alternative to or in conjunction with mediation based methods. We show that this method is still liable to measurement error, but because it has different properties to the CIT it offers several main advantages. First, it uses a formal statistical framework to test for the reliability of the assumed direction of causality. Second, after testing in a comprehensive range of scenarios the MR based approach is less likely to infer the wrong direction of causality compared to CIT, while substantially improving power over CIT in the cases where *d* > 0.

We demonstrate this new method by evaluating the causal relationships of 458 known associations between DNA methylation and gene expression levels using summary level data. The inferred causal direction is heavily influenced by how much measurement error is present in the different assaying platforms. For example, if DNA methylation measures typically have lower or equal measurement error compared to gene expression measures then our analysis suggests that DNA methylation levels would be more often the causal factor in the association. Indeed, previous studies which have evaluated measurement error in these platforms do support this position [42, 43], though making strong conclusions for this analysis is difficult because measurement error is likely to be study specific. We also haven’t accounted for the influence of winner’s curse, which can inflate estimates of the variance explained by SNPs, with higher inflation expected amongst lower powered studies. Using p-values for genetic associations from replication studies will mitigate this problem.

In our simulations we focused on the simple case of a single instrument in a single sample setting with a view to making a fair comparison between MR and the various mediation-based methods available. However, if there is only a single instrument it is difficult to separate between the two competing models of *g* instrumenting a trait which causes another trait, and *g* having pleiotropic effects on both traits independently [44]. Under certain conditions of measurement error the CIT test can distinguish these models. We also note that it is straightforward to extend the MR Steiger approach to multiple instruments, requiring only that the total variance explained by all instruments be calculated under the assumption that they are independent. Multiple instruments can indeed help to distinguish between the causal and pleiotropic models, for example by evaluating the proportionality of the SNP-exposure and SNP-outcome effects [16]. Additionally, if there is at least one instrument for each trait then bi-directional MR can offer solutions to inferring the causal direction [16, 28, 45]. We restricted the simulations to evaluating the causal inference between quantitative traits, but it is possible that the analysis could be extended to binary traits by using the genetic variance explained on the liability scale, taking into account the population prevalence [46]. However, our analysis goes beyond many previous explorations of measurement error by assessing the impacts of both imprecision (noise) and linear transformations of the true variable on causal inference.

Our new method attempts to infer causal directions under the assumption that horizontal pleiotropy (the influence of the instrument on the outcome through a mechanism other than the exposure) is not present. Recent method developments in MR [24, 25] have focused on accounting for the issues that horizontal pleiotropy can introduce when multiple instruments are available, but how they perform in the presence of measurement error remains to be explored. An important advantage that MR confers over most mediation based analysis is that it can be performed in two samples, which can considerably improve power and expand the scope of analysis. However, whether there is a substantive difference in two sample MR versus one sample MR in how measurement error has an effect is not yet fully understood. We have also assumed no measurement error in the genetic instrument, which is not unreasonable given the strict QC protocols that ensure high quality genotype data is available to most studies. We have restricted the scope to only exploring non-differential measurement error and avoided the complications incurred if measurement error in the exposure and outcome is correlated. We have also not addressed other issues pertaining to instrumental variables which are relevant to the question of instrument-exposure specification. One such problem is exposure misspecification, for example an instrument could associate with several closely related putative outcomes, with only one of them actually having a causal effect on the outcome. This problem has shown to be the case for SNPs influencing different lipid fractions, for example [47, 48].

Mediation based network approaches, that go beyond analyses of two variables, are very well established [37] and have a number of extensions that make them valuable tools, including for example network construction. But because they are predicated on the basic underlying principles of mediation they are liable to suffer from the same issues of measurement error. Recent advances in MR methodology, for example applying MR to genetical genomics [49], multivariate MR [48] and mediation through MR [50–52] may offer more robust alternatives for these more complicated problems.

The overarching result from our simulations is that, regardless of the method used, inferring the causal direction using an instrument of unknown biology is highly sensitive to measurement error. With the presence of measurement error near ubiquitous in most observational data, and our ability to measure it limited, we argue that it needs to be central to any consideration of approaches which are used in attempt to strengthen causal inference, and any putative results should be accompanied with appropriate sensitivity analysis that assesses their robustness under varying levels of measurement error.

## Methods

### CIT test

Here we describe how the CIT method [4] is implemented in the R package *R/cit* [18]. Assume an exposure *x* is instrumented by a SNP *g*, and the exposure *x* causes an outcome *y*, as described above. The following tests are then performed:

1. *H*_0_: *cov*(*g*, *y*) = 0; *H*_1_: *cov*(*g*, *y*) ≠ 0; *the SNP associates with the outcome*
2. *H*_0_: *cov*(*g*, *x*|*y*) = 0; *H*_1_: *cov*(*g*, *x*|*y*) ≠ 0; *the SNP associates with the exposure conditional on the outcome*
3. *H*_0_: *cov*(*x*, *y*|*g*) = 0; *H*_1_: *cov*(*x*, *y*|*g*) ≠ 0; *the exposure associates with the outcome conditional on the SNP*
4. *H*_0_: *cov*(*g*, *y*|*x*) ≠ 0; *H*_1_: *cov*(*g*, *y*|*x*) = 0; *the SNP is independent of the outcome conditional on the exposure*

The term in the 4th test can be rewritten as $cov\left(g,y|x\right)=cov\left(g,y-\hat{y}\right)$ where $y-\hat{y}=y-({\hat{\alpha }}_{g}+{\hat{\beta }}_{g}x)$ is the residual of *y* after adjusting for *x*, and *x* is assumed to mediate the association between the SNP and the outcome. The condition in the 4th test is formulated as an equivalence testing problem that is estimated using simulations, comparing the estimate from the data against empirically obtained estimates for simulated variables where the independence model is true (full details are given in [4]). We note here that this approach is liable to fail, even when there is a true causal relationship, when confounders of the exposure and outcome are present, as these will induce collider bias.

If all four tests reject the null hypothesis then it is inferred that *x* causes *y*. The CIT measures the strength of causality by generating an omnibus p-value, *p*_*CIT*_, which is simply the largest (least extreme) p-value of the four tests, the intuition being that causal inference is only as strong as the weakest link in the chain of tests.

Now we describe how we used the CIT method in our simulations. The *cit.cp* function was used to obtain an omnibus p-value. To infer the direction of causality using the CIT method, an omnibus p-value generated by CIT for each of two tests—*p*_*CIT*,*x*→*y*_, was estimated for the direction of *x* causing *y* (Model 1), and for the direction of *y* causing *x*, *p*_*CIT*,*y*→*x*_ (Model 2). The results from each of these methods can then be used in combination to infer the existance and direction of causality. For some significance threshold *α* there are four possible outcomes from these two tests, and their interpretations are as follows:

- If *p*_*CIT*,*x*→*y*_ < *α* and *p*_*CIT*,*y*→*x*_ > *α* then model 1 is accepted
- If *p*_*CIT*,*x*→*y*_ > *α* and *p*_*CIT*,*y*→*x*_ < *α* then model 2 is accepted
- If *p*_*CIT*,*x*→*y*_ > *α* and *p*_*CIT*,*y*→*x*_ > *α* then no evidence for a causal relationship
- If *p*_*CIT*,*x*→*y*_ < *α* and *p*_*CIT*,*y*→*x*_ < *α* then there is potentially confounding (S1 Fig) and no call is made.

For the purposes of compiling simulation results we use an arbitrary *α* = 0.05 value, though we stress that for real analyses it is not good practice to rely on p-values for making causal inference, nor is it reliable to depend on arbitrary significance thresholds [53].

### MR causal test

Two stage least squares (2SLS) is a commonly used technique for performing MR when the exposure, outcome and instrument data are all available in the same sample. A p-value for this test, *p*_*MR*_, was obtained using the *systemfit* function in the R package *R*/*systemfit* [54]. Note that the value of *p*_*MR*_ is identical when using the same genetic variant to instrument the influence of the exposure *x* on the outcome *y*, or erroneously, instrumenting the outcome *y* on the exposure *x*.

The method that we will now describe is designed to distinguish between two models, *x* → *y* or *y* → *x*. Unlike the CIT framework, this approach cannot infer if the true model is *x* ← *g* → *y*. We also assume all genetic effects are additive.

To infer the direction of causality it is desirable to know which of the variables, *x* or *y*, is being directly influenced by the instrument *g*. This can be achieved by assessing which of the two variables has the biggest absolute correlation with *g* (S2 Text), formalised by testing for a difference in the correlations *ρ*_*gx*_ and *ρ*_*gy*_ using Steiger’s Z-test for correlated correlations within a population [55]. It is calculated as

$$Z=\left(Z_{gx}-Z_{gy}\right)\frac{\sqrt{N-3}}{\sqrt{2(1-\rho_{xy})h}}$$

where Fisher’s z-transformation is used to obtain $Z_{g*}=\frac{1}{2}\ln\;(\frac{1+\rho_{g*}}{1-\rho_{g*}})$,

$$h=\frac{1-(frm^{2})}{1-rm^{2}}$$

where

$$f=\frac{1-\rho_{xy}}{2(1-rm^{2})}$$

and

$$rm^{2}=\frac{1}{2}\left(\rho_{gx}^{2}+\rho_{gy}^{2}\right).$$

The *Z* value is interpreted such that

$$Z\;\{\begin{array}{cc} >0, & x\to y\\ <0, & y\to x\\ =0, & x⊥\;\;\;\;⊥y \end{array}$$

and a p-value, *p*_*Steiger*_ is generated from the *Z* value to indicate the probability of obtaining a difference between correlations *ρ*_*gx*_ and *ρ*_*gy*_ at least as large as the one observed, under the null hypothesis that both correlations are identical.

The existence of causality and its direction is inferred based on combining information from the MR analysis and the Steiger test. The MR analysis indicates whether there is a potential causal relationship (*p*_*MR*_), and the Steiger test indicates the direction (*sign*(*Z*)) of the causal relationship and the confidence of the direction (*p*_*Steiger*_). For the purposes of compiling simulation results, these can be combined using an arbitrary *α* = 0.05 value:

- If *p*_*Steiger*_ < *α* and *p*_*MR*_ < *α* and *Z* > 0 then a causal association for the correct model is accepted, *x* → *y*
- If *p*_*Steiger*_ < *α* and *p*_*MR*_ < *α* and *Z* < 0 then a causal association for the incorrect model is accepted, *y* → *x*
- Otherwise if *p*_*Steiger*_ > *α* or *p*_*MR*_ > *α*, neither model is accepted

Note that the same correlation test approach can be applied to a two-sample MR setting. Two-sample MR refers to the case where the SNP-exposure association and SNP-outcome association are calculated in different samples (e.g. from publicly available summary statistics [26, 30]). Here the Steiger test of two independent correlations can be applied where.

$$Z=\frac{Z_{gx}-Z_{gy}}{\sqrt{1/\left(N_{1}-3\right)+1/\left(N_{2}-3\right)}}$$

An advantage of using the Steiger test in the two sample context is that it can compare correlations in independent samples where sample sizes are different. Steiger test statistics were calculated using the *r.test* function in the R package *R/psych* [56].

The Steiger test assumes that there is a causal relationship between the two variables, and that the SNP is a valid instrument for one of them. However it is liable to give incorrect causal directions under some other circumstances. First, some levels of horizontal pleiotropy, where the SNP influences the outcome through some pathway other than the exposure, could induce problems because this is a means by which the instrument is invalid. Second, some differential values of measurement error between the exposure and the outcome could lead to incorrect inference of the causal direction (S2 Text). Third, some levels of unmeasured confounding between the exposure and the outcome could lead to inference of the wrong causal direction (S3 Text).

### Causal direction sensitivity analysis for measurement error

The Steiger test for inferring if *x* → *y* is based on evaluating *ρ*_*gx*_ > *ρ*_*gy*_. However, *ρ*_*gx*_ (or *ρ*_*gy*_) are underestimated if *x* (or *y*) are measured imprecisely. If, for example, *x* has lower measurement precision than *y* then we might empirically obtain *ρ*_*g*,*x*_*o*__ < *ρ*_*g*,*y*_*o*__ because *ρ*_*g*,*x*_*o*__ could be underestimated more than *ρ*_*g*,*y*_*o*__.

As we show in S2 Text it is possible to infer the bounds of measurement error on *x*_*o*_ or *y*_*o*_ given known genetic associations. The maximum measurement imprecision of *x*_*o*_ is *ρ*_*g*,*x*_*o*__, because it is known that at least that much of the variance has been explained in *x*_*o*_ by *g*. The minimum is 0, denoting perfectly measured trait values (the same logic applies to *y*_*o*_). It is possible to simulate what the inferred causal direction would be for all values within these bounds.

To evaluate how reliable, *R*, the inference of the causal direction is to potential measurement error in *x* and *y* we need to predict the values of *ρ*_*gy*_ − *ρ*_*gx*_ for those values of measurement error. We offer two tools in which to do this. First, the user can provide values of measurement error for *x* and *y* and obtain a revised inference of the causal direction. Second, we integrate over the entire range of *ρ*_*gy*_ − *ρ*_*gx*_ values for possible measurement error values, assuming that any measurement error value is equally likely. Across all possible values of measurement error in *x* and *y* we find the volume that agrees with the inferred direction of causality and the volume that disagrees with the inferred direction of causality, and take the ratio of these two values. A ratio *R* = 1 indicates that the inferred causal direction is highly sensitive to measurement error, because equal weight of the measurement error parameter space supports each direction of causality. In general, the *R* value denotes that the inferred direction of causality is *R* times more likely to be the empirical result than the opposite direction (S2 Text).

### Simulations

Simulations were conducted by creating variables of sample size *n* for the exposure *x*, the measured values of the exposure *x*_*o*_, the outcome *y*, the measured values of the outcome *y*_*o*_ and the instrument *g*. One of two models are simulated, the “causal model” where *x* causes *y* and *g* is an instrument for *x*; or the “non-causal model” where *g* influences a confounder *u* which in turn causes both *x* and *y*. Here *x* and *y* are correlated but not causally related. Each variable in the causal model was simulated such that:

$$\begin{array}{ccc} g & ∼ & Binom(2,0.5)\\ x & = & \alpha_{g}+\beta_{g}g+\epsilon_{g}\\ x_{o} & = & \alpha_{mx}+\beta_{mx}x+\epsilon_{mx}\\ y & = & \alpha_{x}+\beta_{x}x+\epsilon_{x}\\ y_{o} & = & \alpha_{my}+\beta_{my}y+\epsilon_{my} \end{array}$$

where non-differential measurement error is represented by a noise (measurement imprecision) term $\epsilon_{m*}∼N\left(0,\sigma_{m*}^{2}\right)$, and measurement bias terms *α*_m*_ and *β*_m*_ for the exposure variable *x* and the outcome variable *y*. Note that following the first section of the Results we no longer include the bias terms for simplicity. We have formulated the non-causal model as:

$$\begin{array}{cc} y & =\alpha_{gy}+\beta_{gy}g+\epsilon_{gy} \end{array}$$

All *α* values were set to 0, and *β* values set to 1. Normally distributed values of ϵ_*_ were generated such that

$$\begin{array}{ccc} cor{\left(g,x\right)}^{2} & = & 0.1\\ cor{\left(x,y\right)}^{2} & = & \left\{0.2,0.4,0.6,0.8\right\}\\ \sigma_{mx}^{2} & = & \left\{0,0.2,0.4,0.6,0.8,1\right\}\\ \sigma_{my}^{2} & = & \left\{0,0.2,0.4,0.6,0.8,1\right\}\\ n & = & \left\{100,1000,10000\right\} \end{array}$$

giving a total of 432 combinations of parameters. Simulations using each of these sets of variables were performed 100 times, and the CIT and MR methods were applied to each in order to evaluate the causal association of the simulated variables. Similar patterns of results were obtained for different values of *cor*(*g*, *x*).

### Applied example using two sample MR

Two sample MR [30] was performed using summary statistics for genetic influences on gene expression and DNA methylation. To do this we obtained a list of 458 gene expression—DNA methylation associations as reported in Shakhbazov et al [39]. These were filtered to be located on the same chromosome, have robust correlations after correcting for multiple testing, and to share a SNP that had a robust cis-acting effect on both the DNA methylation probe and the gene expression probe. Because only summary statistics were available (effect, standard error, effect allele, sample size, p-values) for the instrumental SNP on the methylation and gene expression levels, the Steiger test of two independent correlations was used to infer the direction of causality for each of the associations. The Wald ratio test was then used to estimate the causal effect size for the estimated direction for each association.

All analysis was performed using the R programming language [57] and code is made available at https://github.com/explodecomputer/causal-directions and implemented in the MR-Base (http://wwww.mrbase.org) platform [26].

## Supporting information

S1 Text The influence of measurement error in the exposure on mediation-based estimated. (PDF)

S2 Text Sensitivity analysis for measurement error on the MR Steiger test. (PDF)

S3 Text The influence of unmeasured confounding on the inference of causal directions. (PDF)

S1 Fig Influence of confounding on CIT. Illustrative simulations (*n* = 5000) showing the results from CIT analysis under a model of confounding. Here, the phenotypes *x* and *y* are not causally related, but there is a genetic effect and a confounder both influencing each phenotype. Each point represents a single simulation. Where power is high (when the absolute values of the *x* and *y* axes are large) the CIT returns a significant result (*p* < 0.01) when testing the causal effect of *x* on *y*, and when testing the causal effect of *y* on *x*.
(TIF)

S2 FigInfluence of confounding on MR Steiger.Graph representing the unmeasured confounding parameters that will lead to the MR Steiger test returning the wrong causal direction. Columns of boxes represent different signed values of the observational variance explained between *x* and *y* ($R_{xy}^{2}$).(TIF)
