# Correction: Orienting the causal relationship between imprecisely measured traits using GWAS summary data

**DOI:** 10.1371/journal.pgen.1007149

**Published:** 2017-12-29

**Authors:** Gibran Hemani, Kate Tilling, George Davey Smith

Reference 36 is incorrect. The correct reference is: Wang L, Michoel T (2017) Efficient and accurate causal inference with hidden confounders from genome-transcriptome variation data. PLoS Comput Biol 13(8): e1005703.
