# Supplementary material for: Orienting the causal relationship between imprecisely measured traits using GWAS summary data
Source: PLoS Genet. 2017 Nov 17;13(11):e1007081. doi: 10.1371/journal.pgen.1007081 (PMC5711033; doi:10.1371/journal.pgen.1007081)
Supplement: S1 Text — (PDF) [file pgen.1007081.s001.pdf]

Supplementary materials: Orienting the causal relationship between imprecisely measured traits using GWAS summary data

S1 Text. The influence of measurement error in the exposure on mediation-based estimated

We assume the following model

$$
\begin{aligned}
x &= \alpha_g + \beta_g g + \epsilon_g \\
x_o &= \alpha_{mx} + \beta_{mx} x + \epsilon_{mx} \\
y &= \alpha_x + \beta_x x + \epsilon_x \\
y_o &= \alpha_{my} + \beta_{my} y + \epsilon_{my}
\end{aligned}
$$

where x is the exposure on the outcome y, g is an instrument that has a direct effect on x, x_o is the measured quantity of x, where measurement error is incurred from linear transformation in α_{mx} and β_{mx} and imprecision from ϵ_{mx}, and y_o is the measured quantity of y, where measurement error is incurred from linear transformation in α_{my} and β_{my} and imprecision from ϵ_{my}. Our objective is to estimate the expected magnitude of association between g and y after conditioning on x. Under the CIT, this is expected to be $cov(g, y_o - \hat{y}_o) = 0$ when x causes y, where $\hat{y}_o = \hat{a}_{x_o} + \hat{\beta}_{x_o} x_o$ is the predicted value of y_o using the measured value of x_o.

We can split $cov(g, y_o - \hat{y}_o)$ into two parts, $cov(g, y_o)$ and $cov(g, \hat{y}_o)$.

Part 1

$$
\begin{aligned}
cov(g, y_o) &= cov(g, \beta_{my} y) \\
&= cov(g, \beta_{my} \beta_x x) \\
&= cov(g, \beta_{my} \beta_x \beta_g g) \\
&= \beta_{my} \beta_x \beta_g var(g)
\end{aligned}
$$

Part 2

$$
\begin{aligned}
cov(g, \hat{y}_o) &= cov(g, \hat{\beta}_{x_o} x_o) \\
&= cov(g, \hat{\beta}_{x_o} \beta_{mx} x) \\
&= cov(g, \hat{\beta}_{x_o} \beta_{mx} \beta_g g) \\
&= \hat{\beta}_{x_o} \beta_{mx} \beta_g var(g)
\end{aligned}
$$

Simpifying further

$$
\begin{aligned}
\hat{\beta}_{x_o} &= \frac{cov(y_o, x_o)}{var(x_o)} \\
&= \frac{cov(\beta_{my} y, \beta_{mx} x)}{\beta_{mx}^2 var(x) + var(\epsilon_{mx})} \\
&= \frac{\beta_{mx}\beta_{my} cov(y, x)}{\beta_{mx}^2 var(x) + var(\epsilon_{mx})} \\
&= \frac{\beta_{mx}\beta_{my}\beta_x var(x)}{\beta_{mx}^2 var(x) + var(\epsilon_{mx})}
\end{aligned}
$$

which can be substituted back to give

$$
\begin{aligned}
cov(g, \hat{y}_o) &= \frac{\beta_{my}\beta_x\beta_g var(g) \beta_{mx}^2 var(x)}{\beta_{mx}^2 var(x) + var(\epsilon_{mx})} \\
&= \frac{\beta_{mx}^2 var(x)}{\beta_{mx}^2 var(x) + var(\epsilon_{mx})} \times \beta_{my}\beta_x\beta_g var(g)
\end{aligned}
$$

Finally

$$
cov(g, y_o - \hat{y}_o) = \beta_{my}\beta_x\beta_g var(g) - \frac{\beta_{mx}^2 var(x)}{\beta_{mx}^2 var(x) + var(\epsilon_m)} \times \beta_{my}\beta_x\beta_g var(g)
$$

thus $cov(g, y_o - \hat{y}_o) = 0$ if the measurement imprecision in x_o is $var(\epsilon_m) = 0$. However if there is any imprecision then the condition $cov(g, y_o - \hat{y}_o) = 0$ will not hold.
