# Supplementary material for: Orienting the causal relationship between imprecisely measured traits using GWAS summary data
Source: PLoS Genet. 2017 Nov 17;13(11):e1007081. doi: 10.1371/journal.pgen.1007081 (PMC5711033; doi:10.1371/journal.pgen.1007081)
Supplement: S2 Text — (PDF) [file pgen.1007081.s002.pdf]

Supplementary materials: Orienting the causal relationship between imprecisely measured traits using GWAS summary data

S2 Text. Sensitivity analysis for measurement error on the MR Steiger test

Assuming that either $x \to y$ or $y \to x$, the causal direction can be inferred by evaluating which of $\rho_{g,x}$ and $\rho_{g,y}$ is larger in magnitude. The Steiger test is a hypothesis test that provides a p-value for observing the difference in these correlations under the null hypothesis that they are equal.

Assuming the causal direction is $x \to y$, two stage MR is formulated using the following regression models:

$$x = \alpha_1 + \beta_1 g + e_1$$

for the first stage and

$$y = \alpha_2 + \beta_2 \hat{x} + e_2$$

where $\hat{x} = \hat{\alpha}_1 + \hat{\beta}_1 g$. Writing in scale free terms, $\rho_{g,x}$ denotes the correlation between g and the exposure variable x, and it is expected that $\rho_{g,x} > \rho_{g,y}$ because $\rho_{g,y} = \rho_{g,x}\rho_{x,y}$, where $\rho_{x,y}$ is the causal association between x and y (which is likely to be less than 1).

In the presence of measurement error in x and y, however, the empirical inference of the causal direction will instead be based on evaluating $\rho_{g,x_o} > \rho_{g,y_o}$, which can be simplified:

$$\begin{aligned}
\rho_{g,x_O} &> \rho_{g,y_O} \\
\rho_{g,x}\rho_{x,x_O} &> \rho_{g,y}\rho_{y,y_O} \\
\rho_{g,x}\rho_{x,x_O} &> \rho_{g,x}\rho_{x,y}\rho_{y,y_O} \\
\rho_{x,x_O} &> \rho_{x,y}\rho_{y,y_O}
\end{aligned}$$

In order to assess how reliable the inference of the causal direction is in the presence of measurement imprecision, we can evaluate the range of potential values of measurement error in x and y over which the empirical difference in ρ_{g,x_o} and ρ_{g,y_o} would return the wrong causal direction.

For different values of ρ_{x,x_o}, $\rho_{g,x} = \frac{\rho_{g,x_o}}{\rho_{x,x_o}}$ and $\rho_{g,x_o} \le \rho_{x,x_o} \le 1$. For different values of ρ_{y,y_o}, $\rho_{g,y} = \frac{\rho_{g,y_o}}{\rho_{y,y_o}}$ and $\rho_{g,y_o} \le \rho_{y,x_o} \le 1$.

Call $z = \rho_{g,y} - \rho_{g,x}$ the true difference in the variance explained by the genetic variant in y and x. If $z < 0$ then we infer that $x \to y$. There will be some values of ρ_{x,x_o} and ρ_{y,y_o} that do not alter whether $z < 0$. To evaluate the reliability, R, of the inference of the causal direction with regards to measurement error, the objective is to compare the proportion of the parameter space that agrees with the inferred direction against the proportion which does not:

$$R = \frac{V_{z \ge 0}}{-V_{z<0}}$$

If $R = 1$ then the direction of causality is equally probable across the range of possible measurement error values. If $R > 1$ then R times as much of the parameter space favours the inferred direction of causality. V_z, the total volume of the function (Figure 4), can be obtained analytically by solving:

$$V_z = \int_{\rho_{g,x_o}}^{1} \int_{\rho_{g,y_o}}^{1} \frac{\rho_{g,y_o}}{\rho_{y,y_o}} - \frac{\rho_{g,x_o}}{\rho_{x,x_o}} \;\; d\rho_{y,y_o} d\rho_{x,x_o}$$
$$= \rho_{g,x_o} log(\rho_{g,x_o}) - \rho_{g,y_o} log(\rho_{g,y_o}) + \rho_{g,x_o}\rho_{g,y_o}(log(\rho_{g,y_o}) - log(\rho_{g,x_o}))$$

$V_{z\geq 0}$, the proportion of the volume that lies above the $z = 0$ plane, can also be obtained analytically. The region of this volume is bound by the values of ρ_{x,x_o} and ρ_{y,y_o} where $0 = \rho_{g,y} - \rho_{g,x}$, which can be expanded to $\rho_{y,y_o} = \rho_{g,y_o}\rho_{x,x_o}/\rho_{g,x_o}$. Hence,

$$V_{z\geq 0} = \int_{\rho_{g,x_o}}^{1} \int_{\rho_{g,y_o}}^{\frac{\rho_{g,y_o}\rho_{x,x_o}}{\rho_{g,x_o}}} \frac{\rho_{g,y_o}}{\rho_{y,y_o}} - \frac{\rho_{g,x_o}}{\rho_{x,x_o}} \;\; d\rho_{y,y_o} d\rho_{x,x_o}$$
$$= 2\rho_{g,x_o}\rho_{g,y_o} - 2\rho_{g,y_o} - \rho_{g,y_o} log(\rho_{g,x_o}) - \rho_{g,x_o}\rho_{g,y_o} log(\rho_{g,x_o})$$

Thus $V_{z<0} = V_z - V_{z\geq 0}$.
