# Supplementary material for: Orienting the causal relationship between imprecisely measured traits using GWAS summary data
Source: PLoS Genet. 2017 Nov 17;13(11):e1007081. doi: 10.1371/journal.pgen.1007081 (PMC5711033; doi:10.1371/journal.pgen.1007081)
Supplement: S3 Text — (PDF) [file pgen.1007081.s003.pdf]

Supplementary materials: Orienting the causal relationship between imprecisely measured traits using GWAS summary data

S3 Text. The influence of unmeasured confounding on the inference of causal directions

We have assumed no unmeasured confounding in these simulations. Unmeasured confounding will however have potentially large influences on mediation-based methods for inferring causal directions, and can also adversely influence the estimate of the causal direction for the Steiger test.

Unmeasured confounding in mediation

Including an unmeasured confounder, u, after ignoring intercept terms the exposure x and outcome y variables can be modelled as

$$y = \beta_x x + \beta_{uy} u + \epsilon_x$$
$$x = \beta_g g + \beta_{ux} u + \epsilon_g$$

The observational estimate of the causal effect of x on y, $\hat{\beta}_x$ is obtained from

$$\begin{aligned}\hat{\beta}_x &= cov(x,y)/var(x) \\ &= \frac{\beta_g^2 \beta_x var(g) + \beta_{ux}^2 \beta_x var(u) + \beta_x var(\epsilon_g)}{\beta_g^2 var(g) + \beta_{ux}^2 var(u) + var(\epsilon_g)}\end{aligned}$$

From this it is clear that β_x and $\hat{\beta}_x$ will differ when both β_{uy} and β_{ux} are non-zero. Relating to mediation, where we attempt to test if g associates with y after adjusting y for x, such that

$$\hat{y}^* = \hat{\beta}_x x$$

and

$$\begin{aligned}cov(g, y - \hat{y}^*) &= cov(g, \beta_x x + \beta_{uy} u + \epsilon_x - \hat{\beta}_x x) \\ &= cov(g, (\beta_x - \hat{\beta}_x)(\beta_g g + \beta_u x u + \epsilon_x)) \\ &= (\beta_x - \hat{\beta}_x) var(g)\end{aligned}$$

should any amount of unmeasured confounding exist, therefore, there will remain an association between g and $y|x$, which will introduce errors in inferring causal directions.

Unmeasured confounding in the MR Steiger test

Similarly, we can investigate the extent to which unmeasured confounding will lead to the wrong causal direction between x and y using the MR Steiger test, evaluating the liability for the inequality $cor(g,x)^2 > cor(g,y)^2$ being incorrect. After some algebra

$$cor(g,x)^2 = \frac{\beta_g^2}{\beta_g^2 var(g) + \beta_{ux}^2 var(u) + var(\epsilon_x)}$$

and

$$cor(g,y)^2 = \frac{\beta_x^2 \beta_g^2 var(g)^2}{\hat{\beta}_x^2 \beta_g^2 var(g) + \hat{\beta}_x^2 \beta_{ux}^2 var(u) + \beta_{uy}^2 var(u) + var(\epsilon_y)}$$

S2 fig shows the relationship between the magnitude of the correlations between x, y and the confounder u for a range of $\beta_{xy} = (-2, 2)$, $\beta_{gx} = 0.1$ and a range of confounder effects. The pattern of results were similar for different values of β_{gx}. We note that in most cases for the parameter values explored, where the observational absolute $\hat{R}_{xy}^2$ is less than 0.2, unmeasured confounding will not incur the wrong causal direction in the MR Steiger test.
