# Supplementary figures and images for: Orienting the causal relationship between imprecisely measured traits using GWAS summary data
Source: PLoS Genet. 2017 Nov 17;13(11):e1007081. doi: 10.1371/journal.pgen.1007081 (PMC5711033; doi:10.1371/journal.pgen.1007081)

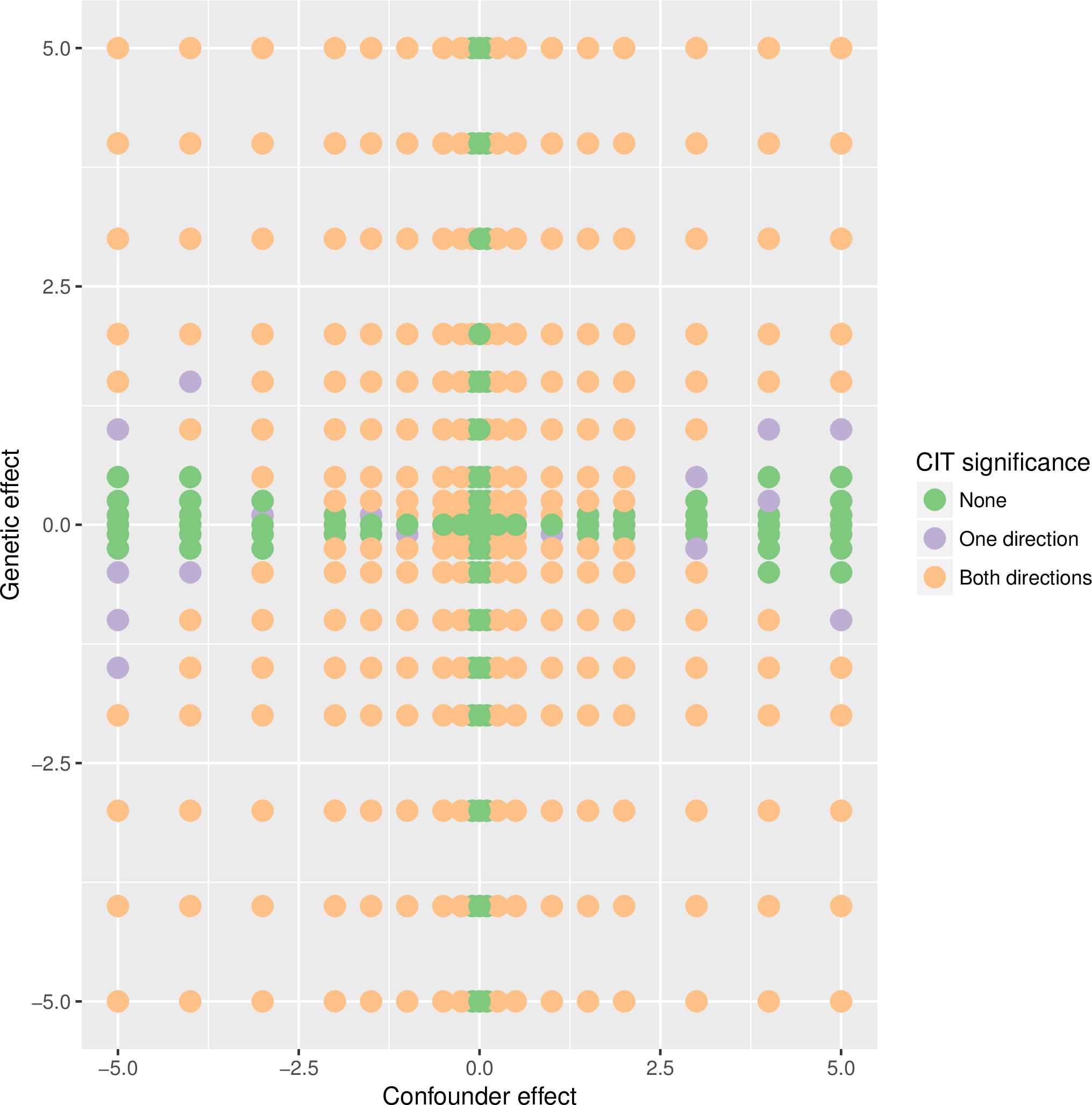

Supplement: S1 Fig — Illustrative simulations (n = 5000) showing the results from CIT analysis under a model of confounding. Here, the phenotypes x and y are not causally related, but there is a genetic effect and a confounder both influencing each phenotype. Each point represents a single simulation. Where power is high (when the absolute values of the x and y axes are large) the CIT returns a significant result (p < 0.01) when testing the causal effect of x on y, and when testing the causal effect of y on x. (TIF) [file pgen.1007081.s004.tif]

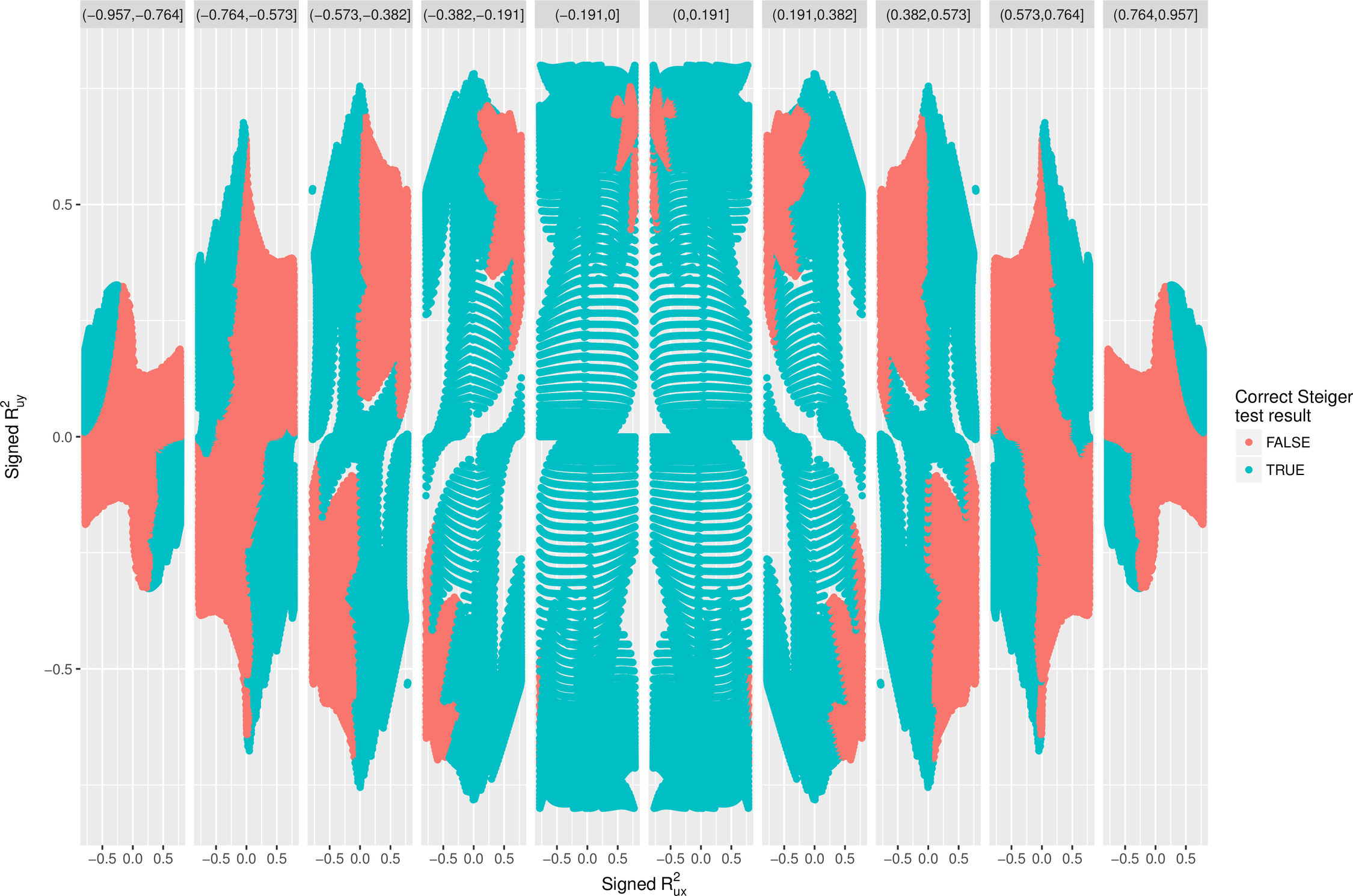

Supplement: S2 Fig — Graph representing the unmeasured confounding parameters that will lead to the MR Steiger test returning the wrong causal direction. Columns of boxes represent different signed values of the observational variance explained between x and y (Rxy2). (TIF) [file pgen.1007081.s005.tif]
